# Numerical data of probabilistic 3D lithological map of Japanese crust

**DOI:** 10.1016/j.dib.2019.104497

**Published:** 2019-09-11

**Authors:** Nozomu Takeuchi, Kenta Ueki, Tsuyoshi Iizuka, Jun Nagao, Akiko Tanaka, Sanshiro Enomoto, Yutaka Shirahata, Hiroko Watanabe, Makoto Yamano, Hiroyuki Tanaka

**Affiliations:** a Earthquake Research Institute, University of Tokyo, Bunkyo-ku, Tokyo, 113-0032, Japan; b Research Institute for Marine Geodynamics, Japan Agency for Marine-Earth Science and Technology, Yokosuka, 237-0061, Japan; c Department of Earth and Planetary Science, University of Tokyo, Bunkyo-ku, Tokyo, 113-0033, Japan; d Geological Survey of Japan, National Institute of Advanced Industrial Science and Technology, Tsukuba, 305-8567, Japan; e Kavli Institute for the Physics and Mathematics of the Universe, University of Tokyo, Kashiwa, 277-8583, Japan; f Department of Physics, University of Washington, Seattle, WA 98195, United States; g Research Center for Neutrino Science, Tohoku University, Sendai, 980-8578, Japan

**Keywords:** Bayesian, Crustal structure, Crustal lithology, Rock composition, Geoneutrinos

## Abstract

The 3D lithological distribution model presented in this data article is related to “Stochastic modeling of 3-D compositional distribution in the crust with Bayesian inference and application to geoneutrino observation in Japan” by Takeuchi et al. (2019) [1]. Our target region is set to the crust and uppermost mantle beneath Japanese main islands and their vicinity. We discretized the target region into 79,968 grid points. We defined 31 rock types; 29 major crustal rock types, plus sediment and mantle. Our lithology model represents a probabilistic distribution map inferred from a seismic tomography model and allows quantitative studies with error estimations, making it fundamentally different from previous models. To enable such quantitative applications, we provide explicit numerical data for the probabilities of the 31 rock types for each grid point. We also provide explicit values of the bulk proportion lithology model at various depths and for the bulk whole crust. Further, a figure of synthetic gravity data is presented to correct a minor error in the related paper [1].

**Table:** Specifications Table

Subject area	*Earth and planetary science*
More specific subject area	*Geochemistry and geophysics*
Type of data	*A spreadsheet (.xlsx), tables, and a figure*
How data was acquired	*Numerical computation*
Data format	*Raw*
Experimental factors	• *We set our target region as Japanese main islands and their vicinity.* • *We adopted an appropriate seismic tomography model of a previous study* *[2]*. • *We adopted the laboratory experimental results of a previous study* *[3]* that measured seismic velocities of each rock type. • *We obtained the prior probabilities of rock types by compiling results from a previous study* *[4]* *that surveyed exposed sections.*
Experimental features	• *We used Bayesian inference to obtain a probability map of rock types from the tomography model, laboratory seismic velocity measurements, and prior probabilities mentioned above.* • *We integrated the probability map to obtain relative frequencies of rock types at various depths and in the whole crust.* • *We simulated a synthetic gravity map from the probability map.*
Data source location	*Crust and uppermost mantle of Japanese main islands and their vicinity*
Data accessibility	*The data are presented within this article.*
Related research article	*[1]**N. Takeuchi, K. Ueki, T. Iizuka, J. Nagao, A. Tanaka, S. Enomoto, Y. Shirahata, H. Watanabe, M. Yamano, H.K.M. Tanaka, Stochastic modeling of 3-D compositional distribution in the crust with Bayesian inference and application to geoneutrino observation in Japan, Phys. Earth Planet. Int. 288 (2019) 37–57.*https://doi.org/10.1016/j.pepi.2019.01.002.

**Table:** 

**Value of the data** • The data explicitly represent the first lithology distribution model with uncertainties that were obtained in a truly statistical and reproducible way. • The data can be used for various quantitative studies in the fields of geochemistry, petrology, geology, geodesy, and particle physics. • The data can be quantitatively compared and/or integrated with other models for other regions if they are obtained by using a similar method. • The data includes a correction to a minor error in a figure in the related paper [1].

## 1 Data

**Fig. 1:**
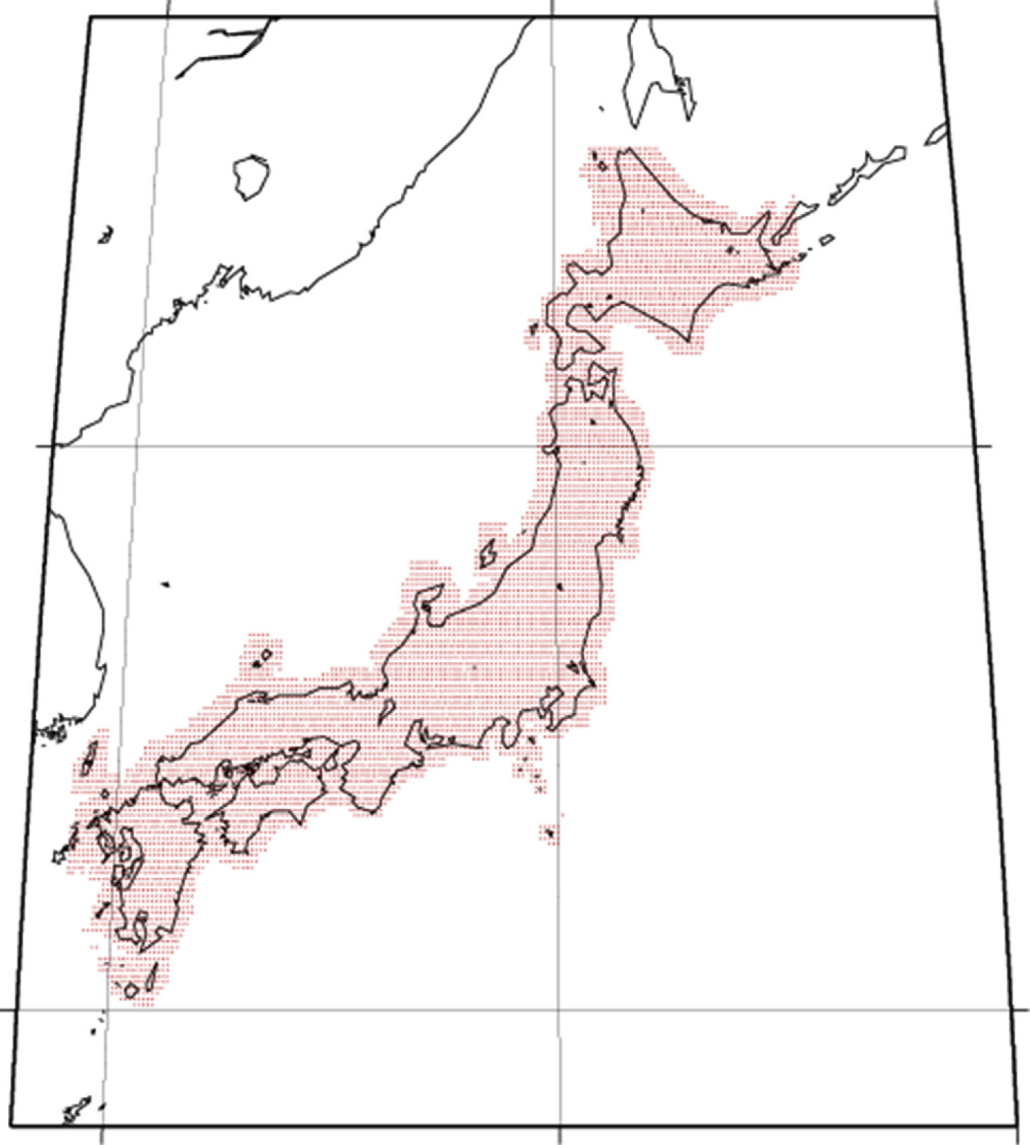
Geographical distribution of $N_{k}\left(=6664\right)$ grid points (red dots) used to represent the lithology distribution model. At each geographical location, we set $N_{l}\left(=12\right)$ grid points in the depth direction.

**Fig. 2:**
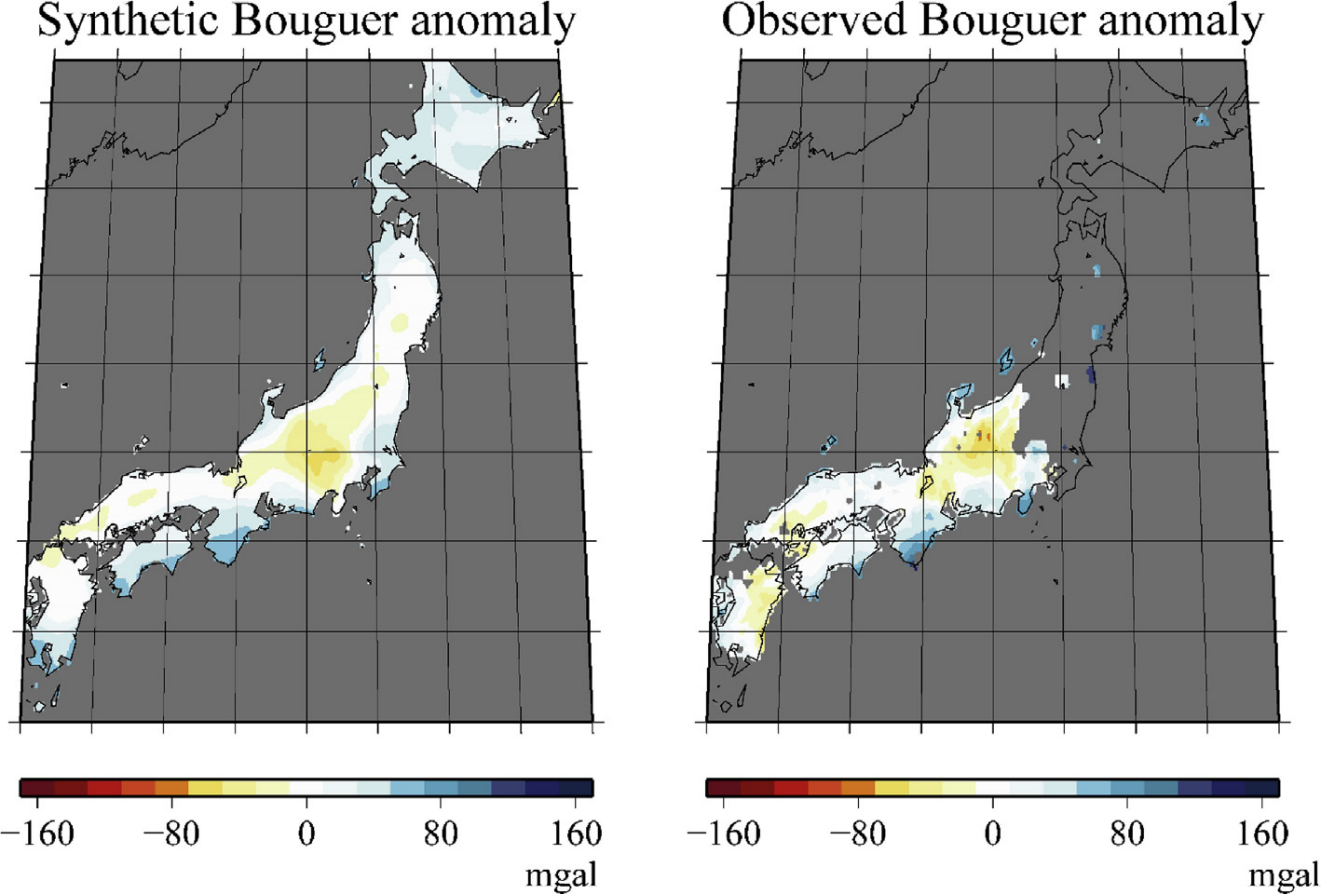
Comparison of the synthetic (left) and observed (right) Bouguer anomalies. The synthetic anomalies are calculated using our inferred probabilistic lithology model.

Our target region is between a depth of 0–50 km beneath the Japanese main islands and their vicinity. The target region is discretized into $N_{k}\left(=6664\right)$ × $N_{l}\left(=12\right)$ grid points in the horizontal and depth directions, respectively (Fig. 1). Their location coincides with the grid points in the tomography model of [2] that was used to construct our model. Fig. 1 shows the geographical location of the grid points, and Table 1 lists the depths of the grid points. We defined 31 rock types: 29 major crustal rock types defined in a previous study [3], plus sediment and mantle. The numerical data (spreadsheet in Appendix A) represents the inferred probabilistic lithology map. Each line in the spreadsheet includes the latitude, longitude, and depth of each grid point and the probabilities of rock types at this point. The probabilities for only 13 of the 31 rock types (listed in Table 2) are presented because those for the other rock types are all zero. Table 1 also lists the relative frequencies of these 13 rock types at each depth. Further, Table 2 lists the bulk crustal proportion model in our target region. Bulk proportions are given for the consolidated crust that excludes the sediment and mantle parts; therefore, values for only 11 rock types are shown. Fig. 2 shows the synthetic Bouguer anomalies computed using our model; it is a revised version of Fig. 4 in the related paper [1], which incorrectly shows the averaged density instead of the synthetic Bouguer anomalies as noted in the caption).

**Table 1:** Relative frequencies (percentage values) of rock types at each depth. The abbreviations used in this table are defined in Table 2.

depth (km)	SED	MGW	SLT	PHY	BGN	GRA	PGR	QSC	MGR	AMP	GAB	GGR	MTL
0.0	2.5	27.5	5.0	20.1	24.4	1.0	5.1	14.5	0.0	0.1	0.0	0.0	0.0
2.5	1.1	24.4	7.0	19.9	26.3	1.5	5.7	13.9	0.0	0.1	0.0	0.0	0.0
5.0	0.1	20.3	8.9	20.8	28.6	1.8	5.8	13.7	0.0	0.1	0.0	0.0	0.0
7.5	0.0	12.7	13.0	20.1	31.5	3.1	7.6	11.7	0.0	0.2	0.0	0.0	0.0
10.0	0.0	8.5	15.1	19.3	32.5	4.5	9.8	9.9	0.1	0.3	0.1	0.0	0.0
15.0	0.0	3.7	11.7	16.4	31.4	6.4	17.3	8.6	1.7	1.7	1.0	0.1	0.0
20.0	0.0	2.2	5.1	10.3	22.3	4.2	16.6	10.9	10.8	8.2	8.4	1.2	0.0
25.0	0.0	0.9	1.0	2.7	6.8	1.0	5.4	9.0	20.0	16.1	30.1	6.7	0.3
30.0	0.0	0.2	0.1	0.3	1.0	0.1	0.8	4.6	12.5	13.4	47.3	12.8	6.8
35.0	0.0	0.0	0.0	0.0	0.1	0.0	0.1	2.0	4.2	6.6	39.3	10.3	37.3
40.0	0.0	0.0	0.0	0.0	0.0	0.0	0.0	0.4	0.5	1.4	13.0	2.9	81.7
50.0	0.0	0.0	0.0	0.0	0.0	0.0	0.0	0.3	0.4	0.8	7.8	1.7	89.0

**Table 2:** Bulk crustal proportion model of rock types in target region.

SED	Sediment	–
MGW	Metagraywacke	6.6%
SLT	Slate	6.0%
PHY	Phyllite	10.7%
BGN	Biotite (Tonalite) Gneiss	18.2%
GRA	Granite-Granodiorite	2.5%
PGR	Paragranulite	7.9%
QSC	Mica Quartz Schist	9.0%
MGR	Mafic Granulite	6.8%
AMP	Amphibolite	6.6%
GAB	Gabbro-norite-troctolite	20.7%
GGR	Mafic Garnet Granulite	5.0%
MTL	Mantle	–

## 2 Experimental design, materials, and methods

We denote the posterior probability of the i^th^ rock type at location $x_{k,l}$ (k and l denote the grid number index in the horizontal and depth directions, respectively) as $P^{\left(x_{k,l}\right)}\left(i|v^{obs}\right)$, where $v^{obs}$ is the observed P velocity in the tomography model referred by this study [2]. The rock types defined here are the 29 major crustal rocks defined in [3] (1≤i≤29), sediment (i=30), and mantle (i=31). Note that we defined sediment (i=30) and mantle (i=31) based not on the consolidating state or chemical composition but as a rock that has P velocity <5.0 km/s and ≥7.5 km/s, respectively. We evaluated the proportion of rock types in the Hidaka crustal section [4] and used it for the prior probability distribution of the lithology in our target region. We used a Bayesian model which updates the prior probability distribution by comparing the seismic velocity of the model lithology with the observed velocity, $v^{obs}$, to obtain the posterior probability distribution, $P^{\left(x_{k,l}\right)}\left(i|v^{obs}\right)$. We referred to a laboratory experiment result of seismic velocity measurements of 29 major crustal rocks [3] for translating $v^{obs}$ into rock types. Only single lithology is assigned to each grid point, while we have shown that the effects of mixed multiple lithology at a grid are marginal (Appendix A.1 and Figure A6 in [1]). The explicit procedures for evaluating $P^{\left(x_{k,l}\right)}\left(i|v^{obs}\right)$ are described in Sections 3.1 and 3.2 in [1]. The explicit values of $P^{\left(x_{k,l}\right)}\left(i|v^{obs}\right)$ are presented in the spreadsheet in Appendix A.

The most important feature of our modeling is that the lithology types are presented by probabilities, $P^{\left(x_{k,l}\right)}\left(i|v^{obs}\right)$, rather than deterministic statement such that we have i-th lithology at the point of $x_{k,l}$. The probabilistic representation allows us to construct probability density functions and thus errors of various physical quantities (such as abundance of radioactive elements, total mass of the crust, and geoneutrino flux) evaluated from the lithology distribution model, while the deterministic statements only allow estimation of central values.

The relative frequency of the i^th^ rock type at the l^th^ depth, $R_{l}\left(i\right)$, in Table 1 was calculated by averaging the probabilities at each depth as follows:

$$R_{l}\left(i\right)=\frac{1}{N_{k}}\overset{N_{k}}{\underset{k=1}{\sum }}P^{\left(x_{k,l}\right)}\left(i|v^{obs}\right)$$

Note that the relative frequencies listed in Table 1 are not for the crustal part but for the whole region including the sediment and mantle. The bulk proportion in the consolidated crust of the i^th^ rock type, X(i), is calculated as

$$X\left(i\right)=\frac{\sum_{l=1}^{N_{l}}R_{l}\left(i\right)\Delta d_{l}}{\sum_{i=1}^{29}\sum_{l=1}^{N_{l}}R_{l}\left(i\right)\Delta d_{l}}\;\;\;\left(1\leq i\leq 29\right)$$

where $\text{Δ}d_{l}$ is the integration thickness for the l^th^ grid point and $\Delta d_{1}$, $\Delta d_{2}$, …, $\Delta d_{12}$ are equal to 1.25, 2.5,2.5, 2.5, 3.75, 5.0, 5.0, 5.0, 5.0, 5.0, 7.5, and 5.0 km, respectively.

The density of the i^th^ rock type at the l^th^ depth, $\rho_{l}\left(i\right)$, has been measured [3] or can be easily evaluated by linear interpolation or extrapolation for the 29 major crustal rock types (1≤i≤29). The density of the sediment and mantle is assumed to be $\rho_{l}\left(30\right)=2.0\times 10^{3}\text{kg}/{\text{m}}^{3}$ and $\rho_{l}\left(31\right)=3.38076\times 10^{3}\text{kg}/{\text{m}}^{3}$, respectively. The expected density $\rho \left(x_{k,l}\right)$ at each grid point location $x_{k,l}$ is calculated as

$$\rho \left(x_{k,l}\right)=\overset{31}{\underset{i=1}{\sum }}\rho_{l}\left(i\right)P^{\left(x_{k,l}\right)}\left(i|v^{obs}\right)$$

The synthetic Bouguer anomalies are calculated by using these quantities (Fig. 2). This figure should have been presented in Fig. 4 in the related paper [1]; however, owing to an editorial error, we instead presented the density averaged over the depth direction, $\underset{l}{\sum }\rho \left(x_{k,l}\right)\Delta d_{l}/\underset{l}{\sum }\Delta d_{l}$. We have now corrected this issue.
